# Integration of Metabolome and Transcriptome Profiling Reveals the Effect of Modified Atmosphere Packaging (MAP) on the Browning of Fresh-Cut Lanzhou Lily (*Lilium davidii* var. *unicolor*) Bulbs during Storage

**DOI:** 10.3390/foods12061335

**Published:** 2023-03-21

**Authors:** Xu Li, Chaoyang Zhang, Xueqi Wang, Xiaoxiao Liu, Xinliang Zhu, Ji Zhang

**Affiliations:** 1 College of Life Science, Northwest Normal University, Lanzhou 730070, China; 2 Institute of New Rural Development, Northwest Normal University, Lanzhou 730070, China; 3 Bioactive Products Engineering Research Center for Gansu Distinctive Plants, Lanzhou 730070, China; 4 Lanzhou Institute of Food and Drug Control, Lanzhou 740050, China

**Keywords:** metabolomics, transcriptomics, Lanzhou lily bulbs, anti-browning, modified atmosphere packaging, fresh-cut fruits and vegetables

## Abstract

The fresh-cut bulbs of the Lanzhou lily (*Lilium davidii* var. *unicolor*) experience browning problems during storage. To solve the problem of browning in the preservation of Lanzhou lily bulbs, we first investigated the optimal storage temperature and gas ratio of modified atmosphere packaging (MAP) of Lanzhou lily bulbs. Then, we tested the browning index (BD), activity of phenylalanine ammonia lyase (PAL), polyphenol oxidase (PPO) and peroxidase (POD), the content of malonaldehyde (MDA) and other physiological activity indicators related to browning. The results showed that the storage conditions of 10% O_2_ + 5% CO_2_ + 85% N_2_ and 4 °C were the best. To further explore the anti-browning mechanism of MAP in fresh-cut Lanzhou lily bulbs, the integration of metabolome and transcriptome analyses showed that MAP mainly retarded the unsaturated fatty acid/saturated fatty acid ratio in the cell membrane, inhibited the lipid peroxidation of the membrane and thus maintained the integrity of the cell membrane of Lanzhou lily bulbs. In addition, MAP inhibited the oxidation of phenolic substances and provided an anti-tanning effect. This study provided a preservation scheme to solve the problem of the browning of freshly cut Lanzhou lily bulbs, and discussed the mechanism of MAP in preventing browning during the storage of the bulbs.

## 1. Introduction

The Lanzhou lily *(Lilium davidii* var. *unicolor)* is a perennial herb of the *liliaceae* family and the bulb is its main edible part. It is mainly distributed in Lanzhou city, Gansu Province, China. According to local government statistics, the planting area of the Lanzhou lily has reached over 700,000 square meters in Lanzhou, and annual production has exceeded 20,000 tons. It has high pharmacological activity and nutritional value; not only does it have polysaccharides, fats, protein, crude fiber, vitamins and other nutrients, but it also contains saponins, alkaloids, flavonoids and other active ingredients in its bulb [1]. Lanzhou lily polysaccharides are known to have anti-inflammation, anti-tumor, and anti-oxidant functions [2,3].

In recent years, with increasing demand for edible, healthy fruits and vegetables, the global market for fresh-cut fruits and vegetables (FFV) has grown rapidly. FFV refers to fresh fruits and vegetables as raw materials after the cleaning, selecting, cutting, molding, preserving and packaging processing of healthy, high-nutrition fruit and vegetable products for convenient consumption [4]. There are also great problems in the production process of FFV, including how to solve tissue softening, cutting surface browning, corruption and other factors caused by mechanical cutting. Browning is a common discoloration phenomenon in the storage of Lanzhou lily bulbs, generally divided into enzymatic browning and non-enzymatic browning. Non-enzymatic browning includes the Maillard reaction, caramel effect, phenolic oxidation condensation reaction and ascorbic acid degradation reaction, etc., without enzymes involved in the browning reaction of fruits and vegetables [5]. Enzymatic browning is due to the oxidation of endogenous phenols into quinones by polyphenols oxidase (PPO) or peroxidase (POD) in the presence of oxygen, and quinones and their derivatives through the further reaction of polymerization to form a relatively insoluble brown pigment called melanin [6]. Phenylalanine ammonia-lyase (PAL) is another key enzyme for enzymatic browning, producing phenols, lignins, flavonoids, anthocyanins, and other secondary metabolites in the phenylalanine pathway [7]. Browning will reduce the sensory quality of FFV and nutritional value and shorten the shelf life, resulting in serious economic losses.

At present, there are many methods to control the browning of FFV, mainly divided into physical preservation technology, chemical preservation technology and biological preservation technology. In physical preservation technology, low-temperature preservation is the most common and effective method. Low-temperature preservation can significantly reduce the activity of PPO and POD in FFV, and also effectively reduce the total phenolic content and activity of PAL, thus slowing down browning. Although chemical preservation technology has a very good anti-browning effect, its safety has been questioned by consumers. For example, the PPO inhibitor sulfite, a color protector commonly used in the food industry, is harmful to human health [8]. In recent years, people have begun to extract substances from plants or fruits and vegetables to use as membranes to prevent browning [9] instead of using synthetic chemical inhibitors. Biological anti-browning techniques mainly consist of the addition of antagonistic anti-bacterial or enzyme engineering, genetic engineering, etc., to extend food shelf life using naturally or artificially controlled microbial flora and (or) the anti-microbial substances they produce [10], or the use of the catalytic effect of enzymes to prevent or eliminate the adverse effects of factors such as microorganisms, oxygen, light and other external factors on the storage process of fruit and vegetables [11], or through relevant genetic engineering means to control the expression of genes related to browning and preservation to achieve the preservation of fruits and vegetables [12].

Since 1970s, MAP has been widely used to maintain and extend the shelf life of the quality of all fresh fruits and vegetables, but it is also increasingly used to extend the shelf life of FFV that are minimally processed in agricultural production [13]; it inhibits the growth of microorganisms by inhibiting the respiration rate of FFV [14], the activity of browning-related enzymes and the synthesis of secondary metabolites, such as phenolics, to reduce the browning of fruits and vegetables [15,16]. At present, there are few studies on the freshness preservation and mechanism of browning in fresh-cut Lanzhou lily bulbs. In recent years, with the rapid development of omics technology, transcriptomics and metabolomics techniques have played an important role in the field of fruit and vegetable post-harvest preservation. In this paper, we will use transcriptomic and metabolomic techniques to study the anti-browning mechanism of fresh-cut Lanzhou lily bulbs in MAP, in order to solve the problem of preserving fresh-cut Lanzhou lily bulbs in storage and transportation, to maximize their nutritional value and extend their shelf life.

## 2. Materials and Methods

### 2.1. Plant Materials

Fresh Lanzhou lily bulbs were collected from Xiguoyuan Town, Qilihe District, Lanzhou City. Fresh lilies with basically uniform size, no pests and diseases, which held tightly and had no visible mechanical damage to the bulbs were selected as the test materials. Before processing test, we removed the outer and inner layers, selected 2~6 layers of fresh Lanzhou lily bulbs of uniform size, rinsed with water and dried the moisture on the surface of the lily.

### 2.2. Temperatures and MAP Preservation

The pre-treated Lanzhou lily bulbs were packed in a PE plastic fresh-keeping box and placed in an incubator (LRH250G, Shanghai Yinze Instrument Equipment Co., Ltd., Shanghai, China) at 20 ± 0.5 °C, 10 ± 0.5 °C, and 4 ± 0.5 °C for 14 d, and each treatment was repeated three times. The browning degree (BD), MDA, PPO, POD and PAL activities of fresh-cut Lanzhou lily bulbs were measured every 2 d.

The pre-treated lily scales were divided into five groups: CK is air control, MAP1: 5% O_2_ + 5% CO_2_ +90% N_2_; MAP2: 10% O_2_ + 5% CO_2_ + 85% N_2_; MAP3: 5% O_2_ + 10% CO_2_ + 85% N_2_; and MAP4: 10% O_2_ + 10% CO_2_ + 80% N_2_. We set N_2_ (purity > 99.99%), O_2_ (purity > 99.99%) and CO_2_ (purity > 99.99%)and used MAP machine (QD-QP-FU-2000, Qingdao Qidong Machinery Co., Ltd., Qingdao, China) packaging. The preservation temperature was 4 °C, the test time was 14 d, and the BD, MDA, PPO, POD and PAL activities of Lanzhou lily bulbs were measured every 2 d.

### 2.3. Determination of BD

According to method of Yang et al. [17], with slight modifications, we weighed 2.0 g of lily sample, added it to a pre-cooled mortar, added distilled water at 0 °C to 4 °C at a ratio of 1:5 (*w/v*), then added a small amount of quartz sand under ice bath conditions to quickly grind it into a uniform size. The slurry was centrifuged at 4 °C and 5000 r/min for 10 min, and the supernatant was taken at a wavelength of 420 nm to measure the absorbance A (ultraviolet spectrophotometer, UV-1000, Labtech, Boston, MA, USA), with distilled water as the blank control. The results were expressed as 10 × A420 for the BD of Lanzhou lily.

BD was determined by following the method of Kan et al. [18] with slight modifications; lily bulb tissue was homogenized at 4 °C using distilled water and centrifuged (5000 r/min (931M-1029-0, Beckman, Brea, CA, USA), 10 min). The absorbance value at 420 nm was determined by a spectrophotometer (Ultraviolet spectrophotometer, UV-1000, Labtech, USA) and BD was expressed as A420 × 10.

### 2.4. Extraction and Assay of PPO, POD and PAL

Lanzhou lily bulb tissue (2.0 g) was homogenized at 4 °C with cold extraction buffer containing phosphate buffer (0.05 mol/L, pH 6.0) and 2% polyvinylpolypyrrolidone (PVPP), and centrifuged (4 °C, 12,000 r/min, 15 min) for the assay of the activities of PPO. Lily bulb tissue (2.0 g) was homogenized at 4 °C with cold extraction buffer containing phosphate buffer (0.05 mol/L, pH 8.0) and 2% polyvinylpolypyrrolidone (PVPP). The homogenates were then centrifuged at 12,000 r/min for 15 min at 4 °C for the assay of the activities of POD. Lily bulb tissue (2.0 g) was homogenized at 4 °C using boric acid-borax buffer (0.1 mol/L, pH 8.8), 1 mmol/L EDTA, 1% polyvinylpyrrolidone PVP and 20 mmol/L mercaptoethanol, then centrifuged (4 °C, 12,000 r/min, 20 min) for the assay of the activities of PAL.

PPO and POD activity was assayed according to method of Yingsanga et al. [19], with slight modifications. For POD activity measure, 0.5 mL of supernatant was mixed with 1.5 mL of phosphate buffer (0.1 mol/L, pH 6.8) and 1 mL catechol solution (0.1 mol/L). Change in absorbance at 410 nm was measured spectrophotometrically. An enzyme activity unit (U) was defined spectrophotometrically as an increase of 0.01 in absorbance per minute per milliliter. For POD activity analysis, 0.5 mL of enzyme extract was added to a reaction mixture consisting of 0.1 mol/L phosphate buffer (pH 8.0), 0.05 mol/L guaiacol solution and 0.5 mol/L H_2_O_2_ (2.5 mL). The change of the mixtion in absorbance at 470 nm was recorded once every 30 s. One unit (U) was defined spectrophotometrically as an increase of 0.01 in absorbance per minute per gram.

PAL activity was assayed according to method of Assis et al. [20], with slight modifications. An amount of 0.5 mL of enzyme extract was added to a reaction mixture consisting of 0.1mol/L boronate-borax buffer solution (PH = 8.8) (3.5 mL) and 20 mmol/L L-phenylalanine (1 mL), then placed in a 40 °C water bath for 50 min. After the incubation, 6 mol/L hydrochloric acid solution (0.1 mL) was added to terminate the reaction immediately. The change of the mixtion in absorbance at 290 nm was recorded. One unit (U) was defined spectrophotometrically as an increase of 0.01 in absorbance per hour per gram.

### 2.5. Determination of Malondialdehyde (MDA)

MDA was assayed according to method of Liu et al. [21], with slight modifications. Lily bulb tissue (2.0 g) was homogenized at 4 °C with cold extraction buffer containing trichloroacetic acid and centrifuged (4 °C, 10,000 r/min, 20 min). For the assay of MDA, 2.0 mL of supernatant was mixed with 2.0 mL 0.67% thiobarbituric acid. Change in absorbance at 532 nm, 600 nm and 450 nm were measured spectrophotometrically.

### 2.6. Transcriptomic Profiling and Data Analysis

The samples from the 1d (bx(MAP)1, ck(control)1), 4d (bx2, ck2), 8d (bx3, ck3) and 14d (bx4, ck4) of the 10 % O_2_ + 5% CO_2_ + 85% N_2_ modified atmosphere preservation experiment at 4 °C were selected, and the low-temperature preservation at 4 °C during the same period was used as the control for transcriptomic analysis. There were three replicate samples per group.

Total RNA of each sample was extracted using TRIzol Reagent/RNeasy Mini Kit (Qiagen)/other kits. Total RNA of each sample was quantified and qualified by Agilent 2100/2200 Bioanalyzer (Agilent Technologies, Palo Alto, CA, USA), NanoDrop (Thermo Fisher Scientific Inc., Waltham, MA, USA). An amount of 1 μg total RNA was used for following library preparation. In order to remove technical sequences, including adapters, polymerase chain reaction (PCR) primers, or fragments thereof, and quality of bases lower than 20, pass filter data of fastq format were processed by Cutadapt (v1.9.1) to become high-quality, clean data. Differential expression analysis used the DESeq2 Bioconductor package (v1.6.3), *p*-adjusted (Padj) of genes was set <0.05 to detect differentially expressed genes. GOSeq (v1.34.1) was used to identify Gene Ontology (GO) terms. KEGG (Kyoto Encyclopedia of Genes and Genomes) was used to enrich significant differential expression genes in KEGG pathways.

### 2.7. Metabolite Profiling and Data Analysis

The samples from the 1d (bx (MAP)1, ck(control)1), 4d (bx2, ck2), 8d (bx3, ck3) and 14d (bx4, ck4) of the 10% O_2_ + 5% CO_2_ + 85% N_2_ modified atmosphere preservation experiment at 4 °C were selected, and the samples were kept at a low temperature of 4 °C during the same period as the control academic analysis. There were six replicate samples per group.

Preparation of sample for Liquid chromatography-Mass spectrometer/Mass spectrometer (LC-MS/MS), the Lanzhou lily bulb samples were frozen with liquid nitrogen and pulverized into powder. Pre-cooled acetonitrile (Cat. No. 271004, Sigma-Aldrich, St. Louis, MO, USA): methanol (Cat. No. 34860, Sigma-Aldrich, St. Louis, MO, USA): water = 2:2:1 (*v/v/v*) was vortexed and ultra-sonicated at low temperature for 30 min, incubated at −20 °C for 10 min, centrifuged at 4 °C for 20 min at 14,000× *g*, and the supernatant was vacuum dried before LC-MS/MS analysis. Samples were kept at −80 °C. We added 150 mL of acetonitrile aqueous solution (acetonitrile: water = 1:1 *v*/*v*) to each sample. The mixture was then vortexed for 1 min before centrifugation at 14,000× *g* for 15 min at 4 °C. Each test sample was combined equally to make a quality control (QC) sample. The analytical method for the QC sample was the same as for the test samples, and the QC sample was inserted after every six test samples to check the instrument’s stability and performance.

UHPLC-Q-Exactive analysis samples were evaluated on a Thermo Scientific^TM^ Vanquish^TM^ Ultra-high performance liquid-chromatography (UHPLC) system (Thermo Fisher Scientific Inc., Waltham, MA, USA). The separation of metabolites was accomplished using a C18 column (Thermo Hypersil Gold C18, 100 mm × 2.1 mm, 1.8 μm, Thermo Fisher Scientific Inc., Waltham, MA, USA) linked to a Thermo Q Exactive (Thermo Fisher Scientific Inc., Waltham, MA, USA) mass spectrometer. The mobile phase consisted of 0.1% formic acid (Cat. No. F0507, Sigma-Aldrich, St. Louis, MO, USA) in water (A) for the positive mode, 5 mM ammonium acetate (Cat. No. 73594, Sigma-Aldrich, St. Louis, MO, USA) in 0.1% formic acid water (A) for the negative mode, as well as acetonitrile (B) under the following gradient conditions: 0–1 min, 1% B; 1–8 min, 1~99% B; 8–10 min, 99% B; 10–10.1 min, 99~1% B; and 10.1–12 min, 1% B. The flow rate was 0.3 mL/min, the column temperature was 35 °C, and the sample injection volume was 2 μL.

LC-MS data were processed by xcmsonline (https://xcmsonline.scripps.edu, accessed on 10 August 2022) using orthogonal partial least-squares discriminant analysis (OPLS-DA) and SIMCA (V. 14.0) software (Sartorius Stedim Data Analytics AB, Umea, Västerbotten County). Variables important in projection (VIP) > 1, Fold change (FC) value (FC > 2 and FC < 0.5) and *t*-test (*p*-adjust < 0.05) were used to determine the different metabolites. KEGG pathway enrichment analysis was used to identify possible biomarker metabolic pathways using MetaboAnalyst 5.0 (http://www.metaboanalyst.ca, accessed on 20 August 2022).

### 2.8. Statistical Analysis

The data were analyzed by a one-way analysis of variance (ANOVA) using SPSS 17.0 (SPSS, Inc., Chicago, IL, USA). Duncan’s multiple range tests with significant differences of *p* < 0.05 were used to compare differences among the mean values. Origin (V. 2022.SR1) software (OriginLab, Northampton, MA, USA) was used to map volcano map of DMs, R language was used for principal component analysis (PCA) and mapped the bubble map of the pathway enrichment analysis.

## 3. Results

### 3.1. Effects of Temperature on the BD, PPO, POD and PAL Enzyme Activities and MDA Content of Lanzhou Lily Bulbs

**Figure 1:**
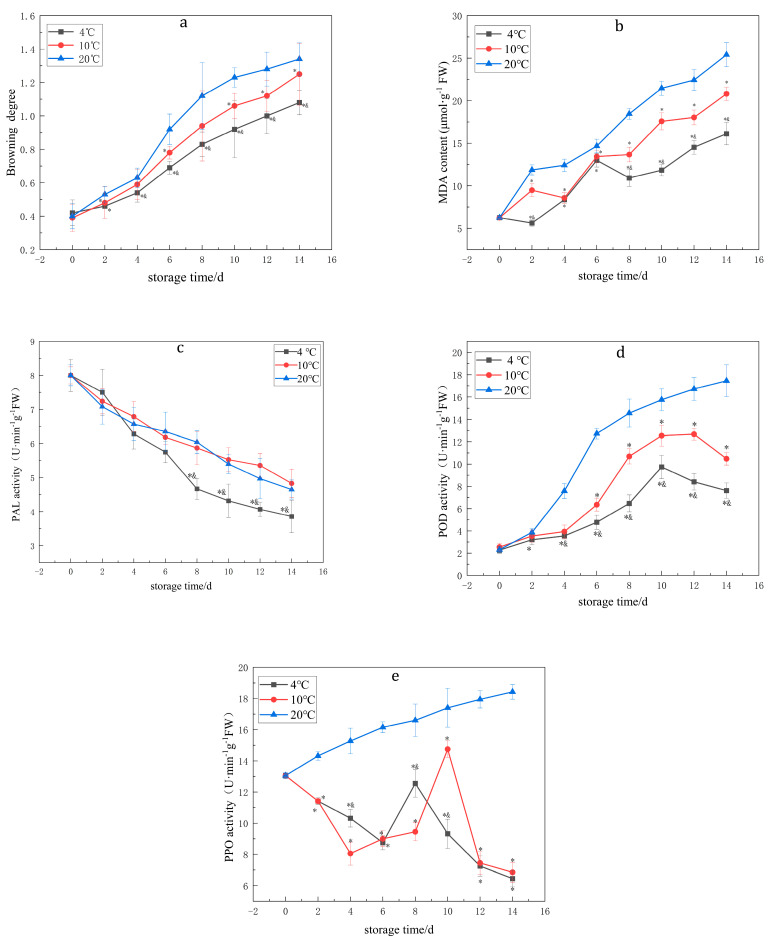
The effects of different temperatures on the BD (**a**); MDA content (**b**); PAL activity (**c**); POD activity (**d**); and PPO activity (**e**) of Lanzhou lily bulbs. (20 °C is the control group and the fresh-keeping groups are 4 °C and 10 °C. *, compared to 20 °C, *p* < 0.05; &, compared to 10 °C, *p* < 0.05).

As shown in Figure 1 and Table 1, low-temperature preservation at 4 °C was beneficial to delay the rise of the BD of Lanzhou lily bulbs, inhibited the activities of related enzymes (PPO, POD, PAL) and browning, and maintained a low MDA content. Therefore, the low temperature of 4 °C can preserve the quality of Lanzhou lily bulbs throughout the storage period.

### 3.2. Effects of MAP on the BD, PPO, POD and PAL Enzyme Activities and MDA Content of Lanzhou Lily Bulbs

**Figure 2:**
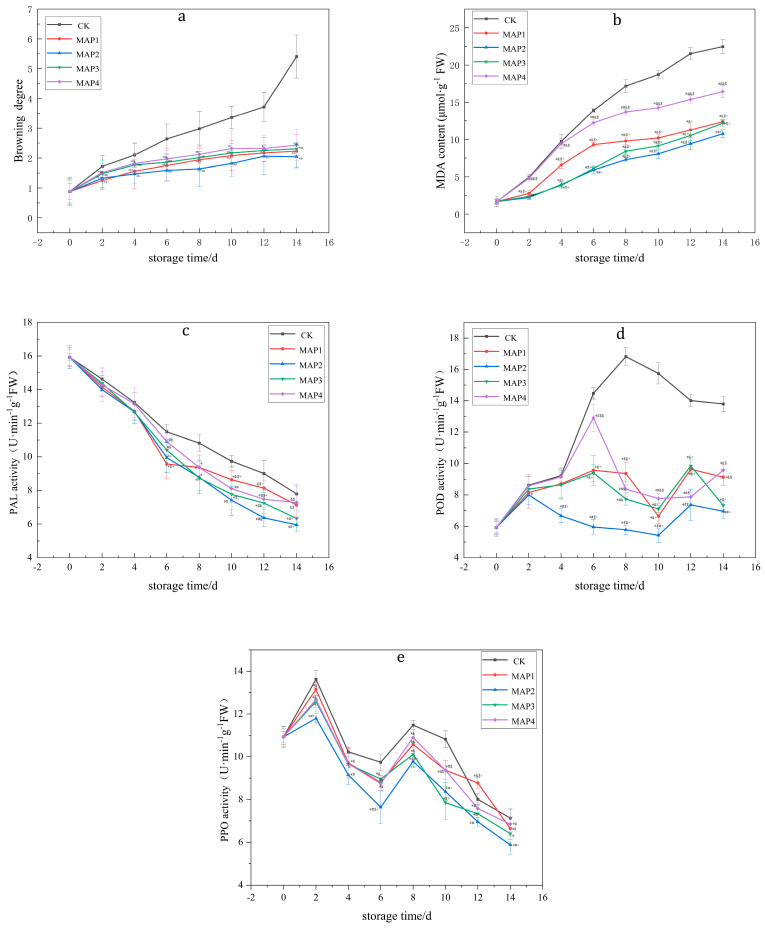
Effects of different proportions of gas on changes in BD (**a**); MDA content (**b**); PAL activity (**c**); POD activity (**d**); and PPO activity (**e**) of Lanzhou lily bulbs. (CK is air control; MAP1: 5% O_2_ + 5% CO_2_ + 90% N_2_; MAP2: 10%O_2_ + 5% CO_2_ + 85% N_2_; MAP3: 5% O_2_ + 10% CO_2_ + 85% N_2_; MAP4: 10% O_2_ + 10% CO_2_ + 80% N_2_. *, compared to CK, *p* < 0.05; #, compared to MAP1, *p* < 0.05; &, compared to MAP2, *p* < 0.05; $, compared to MAP3, *p* < 0.05; +, compared to MAP4, *p* < 0.05).

As shown in Figure 2 and Table 2, the results showed that 10% O_2_ + 5% CO_2_ + 85% N_2_ (MA2) had the most obvious effect on inhibiting the activities of PPO, POD, PAL and the content of MDA, and the BD was the lowest under these conditions.

### 3.3. Sequencing Data Quality Assessment

**Figure 3:**
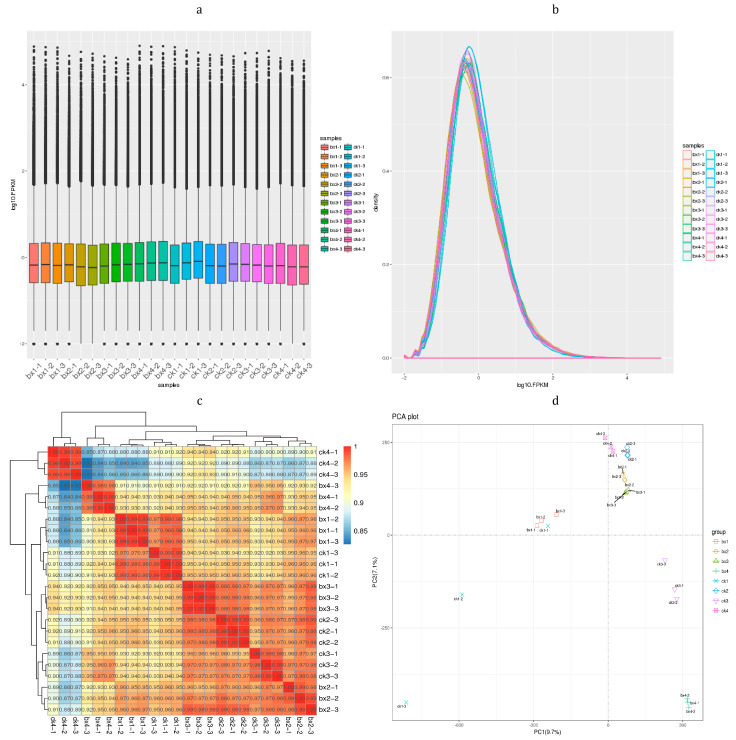
Gene expression analysis. (**a**) Box figure of FPKM. Horizontal is sample ID and ordinate is log_10_^FPKM^; (**b**) FPKM density distribution. Horizontal is log_10_^FPKM^ and ordinate is density; (**c**) Pearson correlation between samples; (**d**) Principal component analysis.

By evaluating the transcriptome data of 24 samples (bx1, ck1 (1d), bx2, ck2 (4d), bx3, ck3 (8d), bx4, ck4 (14d)), RNA-Seq sequencing yielded a total of 43,541,142~55,338, 226 raw reads; Q20 was 97.39~97.78%, Q30 was 92.86~93.83%, and the GC content was 48.44~49.88%, after data filtering. Raw reads were 43,423,484~55,172,454, Q20 was 97.54~97.92%, Q30 was 93.04~94.01%, and the GC content was 48.47~49.93%. The sequencing error rate of each base position was less than 0.5%, the average quality peak value of most base sequences is greater than 30, therefore the sequencing quality met the standard, and further data analysis was performed. The detailed transcriptome data are in the attachment. There are certain differences in the expression of FPKM (fragments per kilobase of transcript per million reads mapped) of genes in each sample, but this difference is not obvious (Figure 3a,b). The correlation analysis (Pearson correlation) showed that the similarity of expression patterns between samples was high, and the samples’ repeatability was good, the test was reliable, and the sample selection was reasonable. At the same time, it can be seen that there are differences in gene expression changes between the preservation treatment and the control group. Moreover, with the prolongation of keep-fresh time, the gene expression also changes (Figure 3c). The results of PCA analysis showed that the Lanzhou lily bulb samples had obvious separation on the 1d, 4d, 8d and 14d, and the difference between the 4d and 8d were small, indicating that DEGs existed at all stages (Figure 3d) (see data in Supplementary Material S1: Data quality assessment.xlsx).

### 3.4. Differentially Expressed Genes (DEGs) Analysis

**Figure 4:**
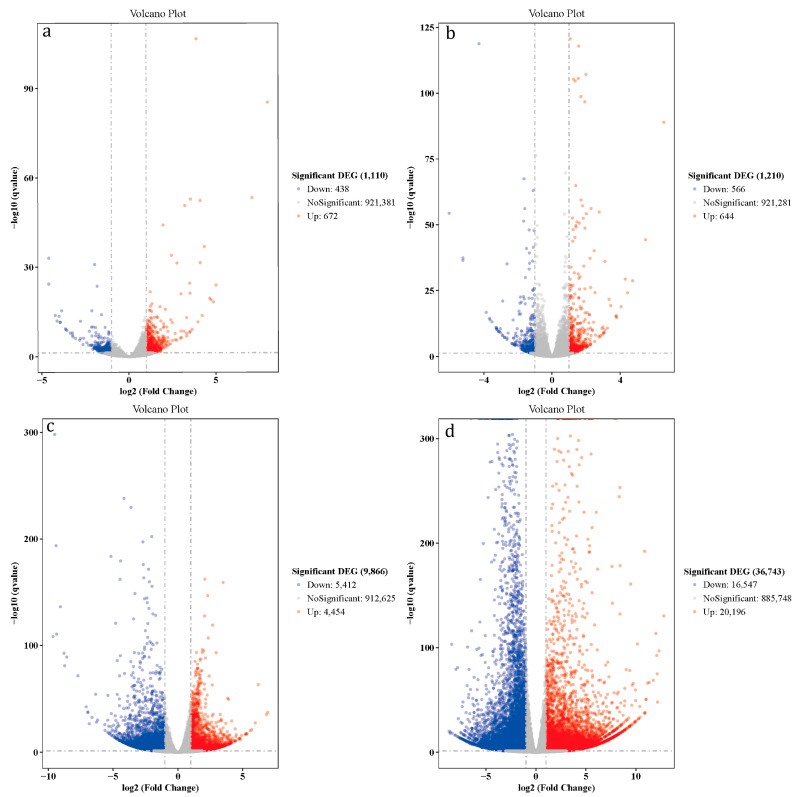
Volcano plots of DEGs. Horizontal is log_2_ (Fold Change) and ordinate is −log10 (*q* value). Red means up-regulation genes, blue means down-regulation genes and grey means no significant genes. (**a**) bx1 vs. ck1; (**b**) bx2 vs. ck2; (**c**) bx3 vs. ck3; (**d**) bx4 vs. ck4.

We set principal standards of |log_2_ (FoldChange)| > 1 and *q* value ≤ 0.05 to acquire DEGs that were significant from the dataset; the results showed that on the 1d (bx1 vs. ck1), the up-regulated and down-regulated DEGs were 438 and 672 (Figure 4a). On the 4d, there were 556 up-regulated and 644 down-regulated DEGs (bx2 vs. ck2) (Figure 4b) and 5412 up-regulated and 4454 down-regulated DEGs on 8d (bx3 vs. ck3) (Figure 4c). The number of up-regulated and down-regulated DEGs were 16,547 and 20,196 on 14d (bx4 vs. ck4) (Figure 4d). In addition, with the prolongation of keep-fresh time, the number of DEGs also increased (see data in Supplementary Material S2: DEGs.xlsx).

### 3.5. GO Enrichment Analysis of DEGs

**Figure 5:**
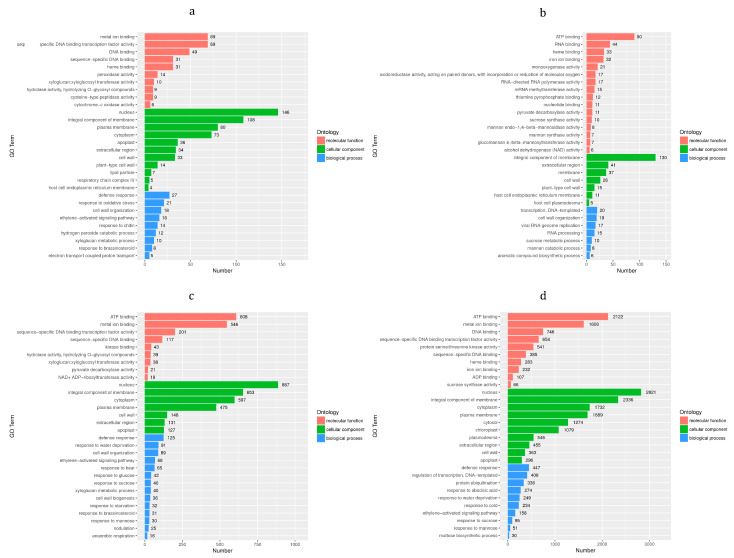
GO enrichment analysis of DEGs. Horizontal is number of DEGs and ordinate is GO term. Different colors were used to distinguish BP, CC and MF. (**a**) bx1 vs. ck1; (**b**) bx2 vs. ck2; (**c**) bx3 vs. ck3; (**d**) bx4 vs. ck4.

GO enrichment analysis was performed on the DEGs (Figure 5), which is used to study the functions of the DEGs at the three levels of Biological Process (BP), Cellular Component (CC) and Molecular Function (MF). On the 1d (Figure 5a), 10 GO terms such as ‘metal ion binging’ and ‘sequence-specific DNA binding transcription factor activity’ were mainly enriched at the MF level. At the CC level, 11 GO terms were mainly enriched such as ‘nucleus, integral component of membrane’, etc. A total of nine GO terms such as ‘defense response and response to oxidative stress’ were enriched at the BP level. On the 4d (Figure 5b), 16 GO terms such as ‘ATP binding and RNA binding’ were mainly enriched at the MF level. A total of seven GO terms such as ‘integral component of membrane’ were mainly enriched at the CC level. A total of seven GO terms such as ‘transcription’, ‘DNA-templated’ and ‘cell wall organization’ were enriched at the BP level. On the 8d (Figure 5c), nine GO terms such as ‘ATP binding’ and ‘metal ion binding’ were mainly enriched at the MF level. The CC level was mainly enriched with seven GO terms such as ‘nucleus’, ‘integral component of membrane’, ‘cytoplasm’, ‘plasma membrane’, etc. The BP level was enriched with 14 GO terms such as ‘defense response’. On the 14d (Figure 5d), 10 GO terms such as ‘ATP binding’ and ‘metal ion binding’ were mainly enriched at the MF level. The CC level was mainly enriched with 10 GO terms such as ‘nucleus’, ‘integral component of membrane’, etc. A total of 10 GO terms such as ‘defense response’, ‘regulation of transcription’, ‘DNA-templated’, etc., were enriched at the BP level (see data in Supplementary Material S3: DEGs-GO analysis.xlsx).

### 3.6. KEGG Enrichment Analysis of DEGs

**Figure 6:**
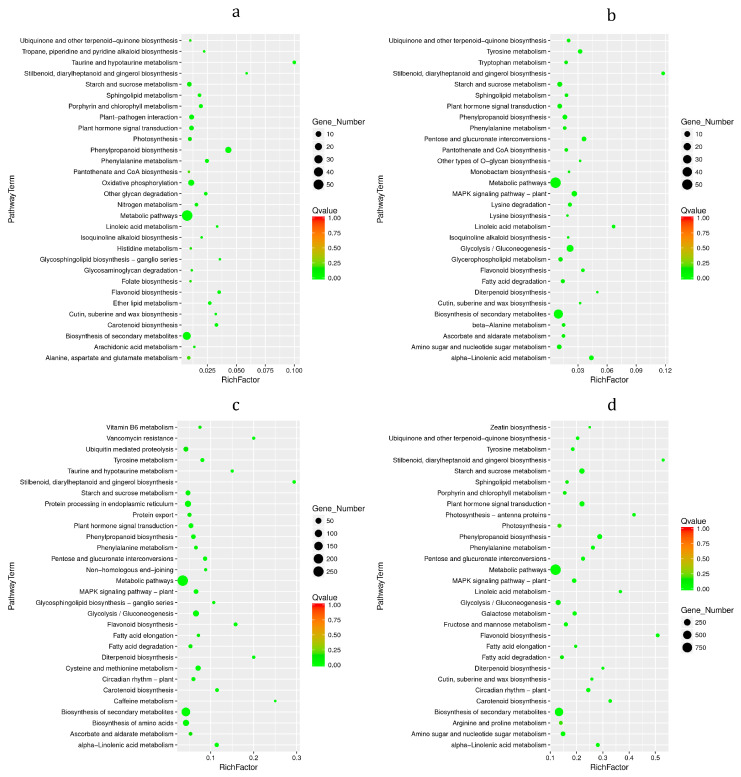
KEGG enrichment analysis of DEGs. Horizontal is number of rich-factor and ordinate is pathway term. The size of the dot represents the number of DEGs. Different colors of dots represent different Qvalues. (**a**) bx1 vs. ck1; (**b**) bx2 vs. ck2; (**c**) bx3 vs. ck3; (**d**) bx4 vs. ck4.

KEGG enrichment analysis was performed on the DEGs (Figure 6). DEGs were mainly enriched in metabolic pathways and biosynthesis of secondary metabolites. On the 1d (Figure 6a), the DEGs were mainly enriched in pathways such as ‘Phenylpropanoid biosynthesis’, ‘Flavonoid biosynthesis’, ‘Taurine and hypotaurine metabolism’ and ‘Stilbenoid, diarylheptanoid and gingerol biosynthesis’. On the 4d (Figure 6b), the DEGs were mainly enriched in pathways such as ‘Phenylpropanoid biosynthesis’, ‘Flavonoid biosynthesis’, ‘Stilbenoid, diarylheptanoid and gingerol biosynthesis’, ‘Linoleic acid metabolism’ and ‘alpha-Linolenic acid metabolism’. On the 8d (Figure 6c), the DEGs were mainly enriched in ‘Phenylpropanoid biosynthesis’, ‘Flavonoid biosynthesis’, ‘alpha-Linolenic acid metabolism’, ‘Stilbenoid, diarylheptanoid and gingerol biosynthesis’, ‘Caffeine metabolism’, ‘Vancomycin resistance and Diterpenoid biosynthesis’ and other pathways. On the 14d (Figure 6d), the DEGs were mainly enriched in ‘Phenylpropanoid biosynthesis’, ‘Flavonoid biosynthesis’, ‘Stilbenoid, diarylheptanoid and gingerol biosynthesis’, ‘alpha-Linolenic acid metabolism’, ‘Carotenoid biosynthesis’, etc., pathways (see data in Supplementary Material S4: DEGs-kegg enrichment.xlsx).

### 3.7. Differential Metabolites (DMs) Analysis

**Figure 7:**
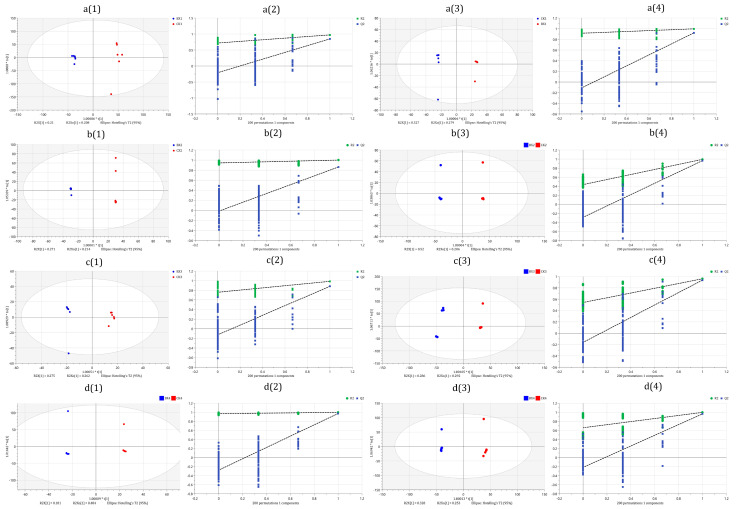
Orthogonal partial least-squares discriminant (OPLS-DA) analysis. (**a(1)**,**b(1)**,**c(1)**,**d(1)**,**a(3)**,**b(3)**,**c(3)**,**d(3)**) were score plot of OPLS-DA model; (**a(2)**,**b(2)**,**c(2)**,**d(2)**,**a(4)**,**b(4)**,**c(4)**,**d(4)**) were overfitting analysis of the OPLS-DA model (two hundred permutations). (**a(1)**–**a(4)**) bx1 vs. ck1; (**b(1)**–**b(4)**) bx2 vs. ck2; (**c(1)**–**c(4)**) bx3 vs. ck3; (**d(1)**–**d(4)**) bx4 vs. ck4.

The OPLS-DA model was used to evaluate the DMs, and the OPLS-DA scores show that each treatment group could be well separated from the control group, which indicated that the metabolites had undergone great changes. As shown in Figure 7, the results of the OPLS-DA model overfitting analysis (200 hypothesis tests) showed that the model was of good quality and was not overfitted. At the same time, VIP was used to identify the main contributing metabolites of the OPLS-DA model.

**Figure 8:**
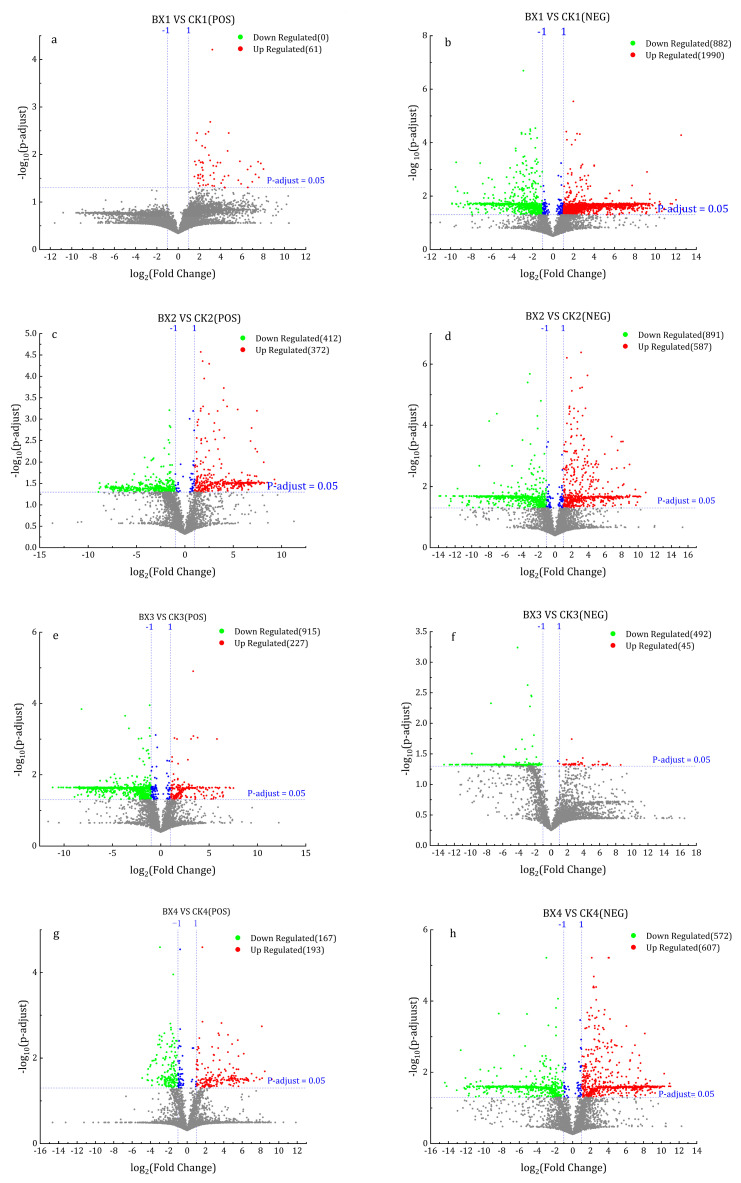
Volcano plot of DMs. Horizontal is log_2_ (Fold Change) and ordinate is −log_10_ (*p*-adjust value). Red means up-regulation DMs, green means down-regulation DMs and grey means no significant DMs. (**a**,**c**,**e**,**g**): DMs in positive ion mode, (**b**,**d**,**f**,**h**): DMs in negative ion mode. (**a**,**b**): bx1 vs. ck1; (**c**,**d**): bx2 vs. ck2; (**e**,**f**): bx3 vs. ck3; (**g**,**h**): bx4 vs. ck4.

In univariate analysis–fold change analysis, DMs were screened with |log_2_ (FoldChange)| > 1 and *p* (adjust) value < 0.05. The results showed that on 1d (bx1 vs. ck1), 61 up-regulated DMs were identified in positive ion mode (Figure 8a), and negative ion mode identified 1990 up-regulated and 882 down-regulated DMs (Figure 8b). On 4d (bx2 vs. ck2), positive ion mode identified 372 up-regulated and 412 down-regulation DMs (Figure 8c), and negative ion mode identified 587 and 891 DMs that were up-regulated and down-regulated (Figure 8d). On 8d (bx3 vs. ck3), positive ion mode identified 227 and 915 up-regulated and down-regulated DMs (Figure 8e), and negative ion mode identified 45 and 492 up-regulated and down-regulated DMs (Figure 8f). On 14d (bx4 vs. ck4), positive ion mode identified 193 and 167 up-regulated and down-regulated DMs (Figure 8g), and negative ion mode identified 607 and 572 up-regulated and down-regulated DMs (Figure 8h) (see data in Supplementary Material S5: DMs.xlsx).

### 3.8. KEGG Enrichment Analysis of DMs

**Figure 9:**
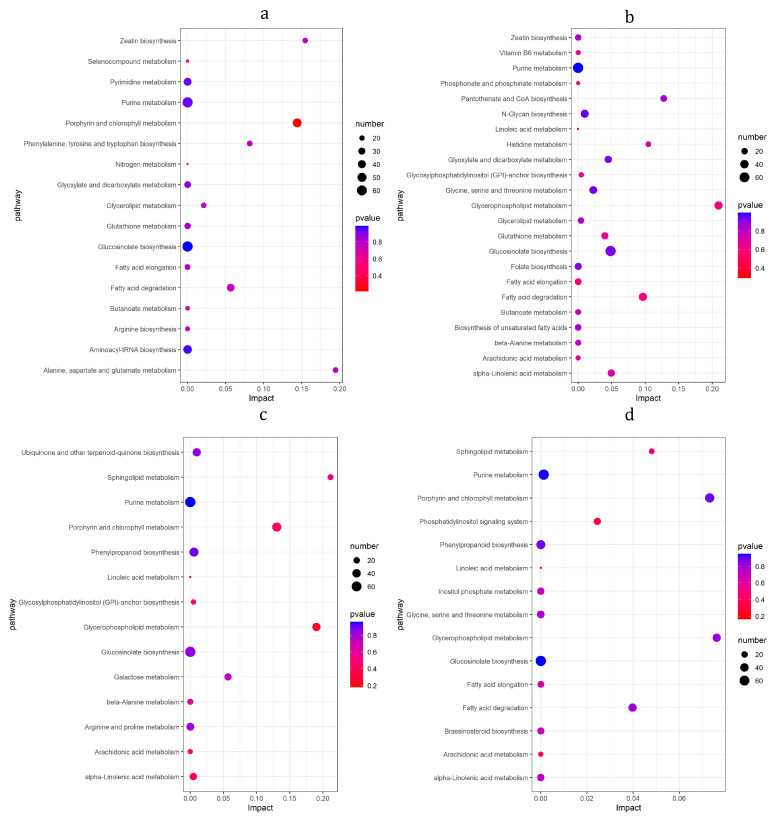
KEGG enrichment analysis of DMs. Horizontal is impact and ordinate is pathway. The size of the dot represents the number of DMs. Different colors of dots represent different *p*-adjust values. (**a**) bx1 vs. ck1; (**b**) bx2 vs. ck2; (**c**) bx3 vs. ck3; (**d**) bx4 vs. ck4.

The results of the KEGG enrichment analysis of DMs (Figure 9) showed that on 1d (Figure 9a), the DMs were mainly enriched in ‘Porphyrin and chlorophyll metabolism’, ‘Zeatin biosynthesis’ and ‘Alanine, aspartate and Glutamate’. On the 4d (Figure 9b), the DMs were mainly enriched in ‘Glycerophospholipid metabolism’, ‘Pantothhenate and CoA biosynthesis’, ‘Histidine metabolism’ and ‘Fatty acid degradation’. On the 8d (Figure 9c), the DMs were mainly enriched in the metabolic pathways such as ‘Sphingolipid metabolism’, ‘Glycerophospholipid metabolism’ and ‘Porphyrin and chlorophyll metabolism’, ‘Phenylpropanoid biosynthesis’ and ‘alpha-linolenic acid metabolism’. On the 14d (Figure 9d), the DMs were mainly enriched in metabolic pathways such as ‘Sphingolipid metabolism’, ‘Glycerophospholipid metabolism’, ‘Porphyrin and chlorophyll’, ‘Fatty acid degradation’ ‘Phosphatidylinositol signaling system’, ‘Phenylpropanoid biosynthesis’, and ‘alpha-linolenic acid metabolism’ (see data in Supplementary Material S6: DMs-kegg enrichment.xlsx).

### 3.9. KEGG Pathway of Differential Gene and Metabolite Co-Enrichment Analysis

**Figure 10:**
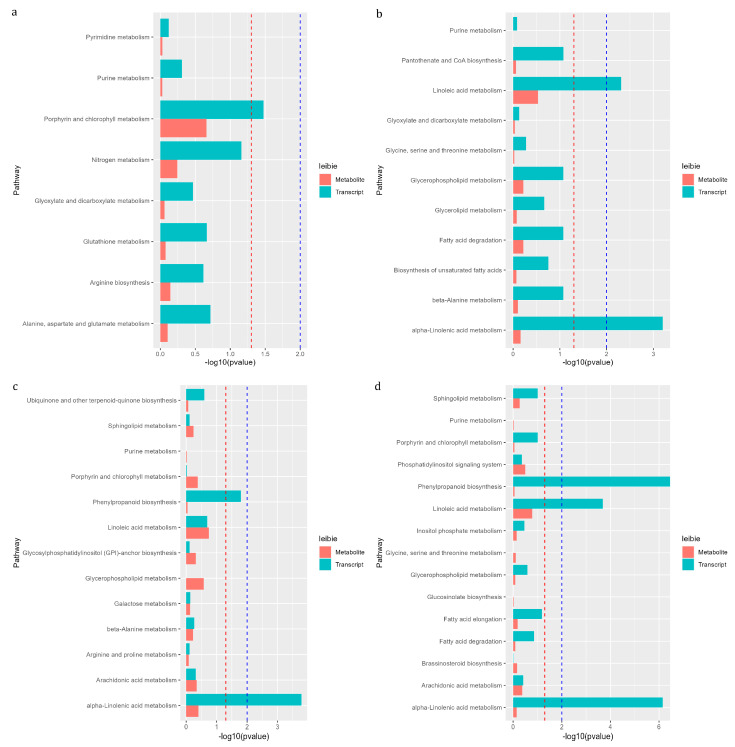
Pathways with simultaneous annotation of DEGs and DMs. Horizontal is −log_10_ (*p*-value) and ordinate is pathway name. Red leibie is metabolite, blue leibie is transcript, red line means *p* value < 0.05 and blue line means *p* value < 0.01. (**a**) bx1 vs. ck1; (**b**) bx2 vs. ck2; (**c**) bx3 vs. ck3; (**d**) bx4 vs. ck4.

To further explore the interaction between DEGs and DMs, we performed a co-enrichment analysis of transcriptome and metabolome. As shown in Figure 10, on the 1d, there were 8 enriched pathways for DEGs and DMs, mainly including ‘Porphyrin and chlorophyll metabolism’ and ‘Nitrogen metabolism’. On the 4d, there were 11 enriched pathways, mainly including ‘Phenylpropanoid biosynthesis’ and ‘Linoleic acid metabolism’. On the 8d, a total of 13 pathways were enriched, mainly including ‘Phenylpropanoid biosynthesis’, ‘Linoleic acid metabolism’ and ‘alpha-Linoleic acid metabolism’. On the 14d, there were a total of enriched 15 pathways, mainly including ‘Phenylpropanoid biosynthesis’, ‘Linoleic acid metabolism’ and ‘alpha-Linolenic acid metabolism’ (Figure 10).

### 3.10. Changes of Metabolites and Genes

**Figure 11:**
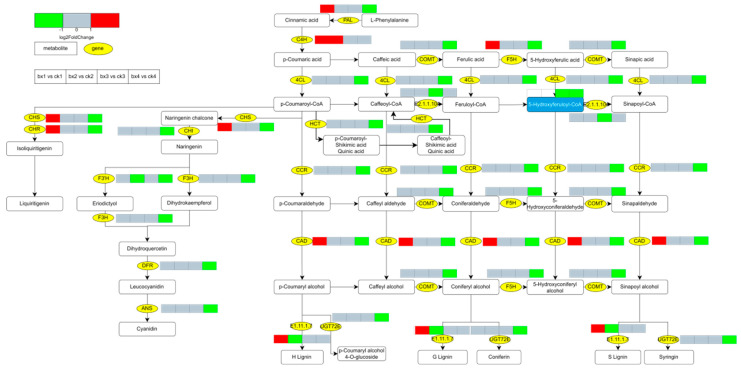
Expression profiles of genes and metabolites involved in ‘Phenylpropanoid biosynthesis’, ‘Flavonoid biosynthesis’ and ‘Stilbenoid, diarylheptanoid and gingerol biosynthesis’. The yellow oval pattern represents the gene and rounded rectangles represent metabolites. Metabolites in blue box were annotated and metabolites in white font were annotated. Four continuous boxes indicate the different fold changes of DEGs or DMs in bx1 vs. ck1; bx2 vs. ck2; bx3 vs. ck3; and bx4 vs. ck4, respectively. Red indicates up-regulated (log_2_foldchange > 1), green indicates down-regulated (log_2_ foldchange < −1), grey indicates no significant change (−1 ≤ log2foldchange ≤ 1), and white indicates the metabolites or genes that were not annotated. 4CL, 4-coumarate: CoA ligase; PAL, Phenylalanine ammonia lyase; CAD, Cinnamoyl alcohol dehydrogenase; C4H, Cinnamic acid 4-hydroxylase; CHS, Chalcone synthase; CHR, Chalcone reductase; CHI, CHS-chalcone isomerase; HCT, hydroxycinnamoyl transferase; DFR, dihydroflavonol 4-reductase; CCR, cinnamoyl CoA reductase; HCT, shikimate hydroxycinnamoyl transferase; F3H, flavanone 3 hydroxylase; F3′H, flavanone 3′-hydroxylase; F5H, flavanone 5 hydroxylase; COMT, caffeic acid O-methyltransferase; ANS, anthocyanidin synthase.

**Figure 12:**
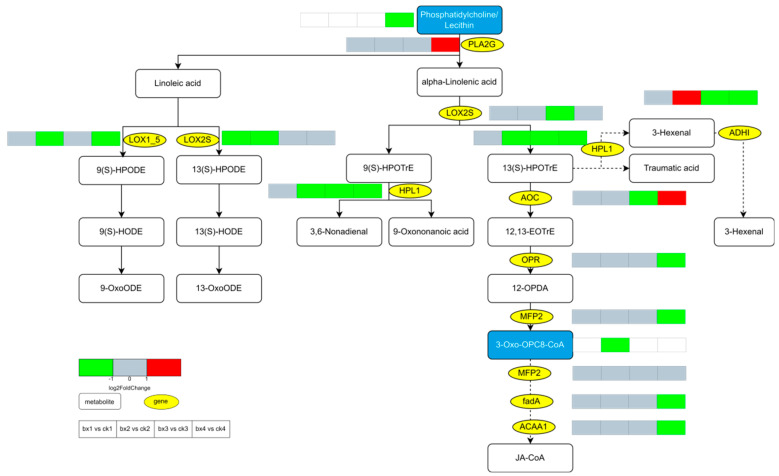
Expression profiles of genes and metabolites involved in linoleic acid metabolism and alpha-linolenic acid metabolism. The yellow oval pattern represents the gene and rounded rectangles represent metabolites. Metabolites in blue box were annotated and metabolites in white font were annotated. Four continuous boxes indicate the different fold changes of DEGs or DMs in bx1 vs. ck1; bx2 vs. ck2; bx3 vs. ck3; and bx4 vs. ck4, respectively. Red indicates up-regulated (log2foldchange > 1), green indicates down-regulated (log2foldchange < −1), grey indicates no significant change (−1 ≤ log2foldchange ≤ 1) and white indicates the metabolites or genes that were not annotated. PLA2G, Phospholipase A2 Group; HPL1, hydroperoxide lyase l; LOX, lipoxygenase; ADH, alcohol dehydrogenase; AOC, allene oxide cyclase; OPR, 12-oxo-phytodienoic acid reductase; MFP2, enoyl-CoA hydratase/3-hydroxyacyl-CoA dehydrogenase; ACAA1, acetyl-CoA acyltransferase 1.

The DEGs and DMs involved in the browning of Lanzhou lily bulbs mainly affected ‘Phenylpropanoid biosynthesis’, ‘Flavonoid biosynthesis’ and ‘Stilbenoid, diarylheptanoid and gingerol biosynthesis’. Lipid metabolism, including ‘linoleic acid metabolism’ and ‘alpha-linolenic acid metabolism’, also were affected. On the 1d, the genes involved in the phenylpropane pathway, such as PAL, cinnamoyl alcohol dehydrogenase (CAD), Cinnamic acid 4-hydroxylase (C4H), Peroxidases, Chalcone synthase (CHS) and Chalcone reductase (CHR) were up-regulated. On the 14d, the main genes involved in the phenylpropane pathway were almost all down-regulated, and genes in the linoleic acid metabolism and alpha-linolenic acid metabolism were also down-regulated (Figure 11 and Figure 12).

## 4. Discussion

Appearance and texture are two fundamental factors that determine the acceptability of FFV [22]. Browning and softening during post-harvest storage cause the appearance quality of Lanzhou lily bulbs to decline, thus affecting consumers’ acceptability and purchasing power. Mechanical damage (fresh cutting) triggers increased respiratory rate and ethylene production in plant tissues [23], destruction of membrane integrity [24] and a large number of physiological and biochemical reactions such as the biosynthesis of secondary metabolites [25].

The integrity of the cell membrane is one of the key factors affecting the browning of fruits and vegetables, mechanical-damage-enhanced membrane lipid peroxidation and lost membrane integrity [26]. Lipids are the basic components of cell membranes. The escape of phenolic substances produces a browning of the tissues under the pro-browning action of Polyphenol oxidase (PPO) [27]. Studies show that linolenic acid and linoleic acid play important roles in fruit browning [28], especially in reducing the proportion of unsaturated fatty acids, which result in the browning of fruits and vegetables [29]. A delayed reduction in linoleic acid and linoleic acid content and increased proportion of unsaturated fatty acid/saturated fatty acid in the fruit can help reduce browning inside the fruit [30,31]. Transcriptomics and metabolomics data showed that key enzymes for the synthesis of linolenic acid and linoleic acid, Phospholipase A2 Group (PLA2G), were up-regulated later in MAP. Linolenic acid and linoleic acid are the main substrates of Lipoxygenase (LOX), so when the activity of lipoxygenase LOX is low, and the metabolic pathway downstream of linolenic acid and linoleic acid metabolism is inhibited, high levels of linolenic acid and linoleic acid are maintained, which inhibits browning. Saquet et al. [32] pointed out that after ‘Conference’ pears were treated by MAP, the relative content of unsaturated fats (such as linolenic acid and linoleic acid) were maintained, the integrity of cell membranes was maintained, and the browning reaction was inhibited. Lin et al. [33] found that the peel of Longan (*Dimocarpus longan Lour.*) treated with hydrogen peroxide (H_2_O_2_) had a higher browning index, higher LOX activity and a lower relative content ratio of unsaturated fatty acids (linoleic acid, linolenic acid)/saturated fatty acids. The cell membrane permeability increases, and the integrity of the membrane structure is lost. MDA can be used as a biochemical marker of membrane structural integrity. LOX catalyzes the oxidation of polyunsaturated fatty acids to conjugated diene and malondialdehyde (MDA); once accumulated, MDA can further damage cell membranes. Dhindsa et al. [34] Gao et al. [35] and Jiang et al. [36] found that the integrity of the membrane was lost during the browning process of fresh-cut lotus root in the control group, and the content of MDA increased in his browning model study. Dokhanieh et al. [37] found that after fresh-cut pomegranate arils were stored at 4 °C for 12 d, browning occurred simultaneously with the accumulation of MDA and H_2_O_2_ and, after hot salicylic acid treatment, the accumulation of MDA was reduced and browning was delayed, which is consistent with our experimental results in this study showing that the MAP reduced the MDA accumulation in Lanzhou lily bulbs. To sum up, this indicates that the MAP may inhibit the browning of Lanzhou lily bulbs by delaying the decrease of the ratio of unsaturated fatty acid/saturated fatty acid content in the cell membrane, further inhibiting membrane lipid peroxidation and maintaining the integrity of the cell membrane of the lily bulbs.

Notably, the biosynthesis of secondary metabolites is another important factor affecting the browning of fruits and vegetables, among which phenylpropane metabolism is an important pathway [38]. Phospholipase (PAL) is an important enzyme of the phenylpropane pathway, catalyzing the formation of L-phenylalanine to trans-cinnamic acid, and then, through a series of biochemical reactions to generate various phenolic substances, anthocyanins, lignin, alkaloids and other substances [39,40], these metabolites provide a precursor substance for oxidative browning. Usually after the plant epidermis is broken (freshly cut), PAL is expressed in large quantities. After the integrity of the cell membrane is impaired, phenolic substances in the vacuole leak, browning-related enzymes and substrates come into contact with each other, and phenolic substrates oxidize to form browning products [41]. In addition, PPO is closely related to tissue browning in fruits and vegetables [42], because PPO can oxidize phenols to quinones under O_2_ catalysis, forming browning products [43]. PPO oxidizes phenols to produce H_2_O_2_, then uses H_2_O_2_ as an electron acceptor to catalyze the oxidation of phenols to form browning products [44]. Physiological experiments have shown that PAL activity, PPO activity and POD activity were at lower levels in the late stage of MAP compared with the control; these results indicate that MAP may inhibit the oxidation reaction of phenolic substances leaking from fresh-cut lily bulbs by inhibiting the activity of PAL, PPO and POD, and further inhibit the formation of browning products. This is similar to the conclusions of numerous studies [41]. High-pressure carbon dioxide (HPCD) treatment of fresh-cut lettuce has shown that HPCD can effectively inhibit PPO and PAL activity [45]. Eugenol emulsions (EUG) on fresh-cut Chinese water chestnut (CWC) showed that EUG reduced the content of phenols and inhibited the browning reaction by reducing the activity of PPO, POD and, especially, PAL enzymes. It is worth noting that phenylpropanoid biosynthesis, flavonoid biosynthesis, stilbenoid, diarylheptanoid and gingerol biosynthesis are closely related to post-harvest disease resistance and color formation of fruits and vegetables. Studies have shown that key genes in the ‘carotenoid biosynthesis’ pathway, ‘flavonoid biosynthesis’ pathway and ‘stilbenoid, diarylheptanoid and gingerol biosynthesis’ pathway in fresh-cut yam are significantly upregulated during yellowing after storage [46]; in the fresh-cut easy-to-brown potato variety YS505, the phenylpropanoid biosynthesis pathway was activated [47]. In addition, it is common for mechanical damage to induce the synthesis and accumulation of phenolic compounds in FFV [48,49]; in order to defend and heal damage, plants quickly synthesize phenolic substances, especially phenolic anti-oxidants, in a short period of time [50]. Many studies have also shown that after damage to different types of freshly cut fruits and vegetables, reactive oxygen species (ROS) were produced rapidly and the content of phenolic compounds and anti-oxidant activity is significantly increased [51].

In this study, omics results showed that the key genes PAL, CAD, C4H Peroxidases, CHS and CHR in phenylpropanoid biosynthesis, flavonoid biosynthesis, stilbenoid, diarylheptanoid and gingerol biosynthesis were up-regulated in the early stage of MAP. There is another possible reason that, compared with the air control, the MAP group gas composition ratio changes, further activating the stress response and enhancing the synthesis pathway of phenolic compounds, but there are few studies in this regard, and the specific mechanism remains to be discussed. It is assuring that the key genes of phenylpropanoid biosynthesis, flavonoid biosynthesis, stilbenoid, diarylheptanoid and gingerol biosynthesis were down-regulated in the post-MAP period (14 d). Studies have shown that low and high O_2_/CO_2_ atmospheres can reduce the respiration rate and browning of fresh-cut potatoes [52]. Similarly, high-pressure carbon dioxide (HPCD) treatment displayed low phenylpropanoid metabolism pathway activity in fresh-cut Chinese water chestnut (CWC) [53]. From this, we speculate that MAP inhibits the synthetic accumulation of phenols and the browning caused by their oxidation in the fresh-cut Lanzhou lily bulb.

The mechanism of anti-browning MAP technology in the preservation of Lanzhou lily bulbs was preliminarily studied through transcriptomics and metabolomics data, and the key genes affecting browning and the key secondary metabolites produced during browning were found. The signal regulatory pathways related to anti-browning were also revealed in this study. However, the continuation of this research needs to verify the anti-browning mechanism of key genes using more molecular biology technology, and verify key secondary metabolites by targeted metabolomics and molecular biology technology, so as to better reveal the anti-browning mechanism of MAP in the preservation process of Lanzhou lily bulbs. This study provided theoretical value and data support for the preservation of fresh-cut fruits and vegetables.

## 5. Conclusions

1. Fresh-cut Lanzhou lily bulbs have the lowest browning index, a good quality and good appearance under the conditions of MAP of 10% O_2_ + 5% CO_2_ + 85% N_2_ and 4 °C.
2. MAP reduces the activity of PAL, PPO, POD and the content of MDA.
3. The mechanism by which MAP inhibits the browning of fresh-cut Lanzhou lily bulbs may be that it retards the reduction in the ratio of unsaturated fatty acids to saturated fatty acids in the cell membrane of the bulbs. Specifically, MAP inhibits the lipid peroxidation of the membrane to maintain the integrity of the cell membrane, and probably inhibits the metabolic pathways of ‘Phenylpropanoid biosynthesis’, ‘Flavonoid biosynthesis’ and ‘Stilbenoid, diarylheptanoid and gingerol biosynthesis’ and the expression of their key enzyme genes, thus inhibiting the oxidation of phenolic substances.

## Figures and Tables

**Table 1:** The effects of different temperatures on the BD, MDA content, PAL activity, POD activity and PPO activity of Lanzhou lily bulbs.

Items	Gruops	0d	2d	4d	6d	8d	10d	12d	14d
BD	4 °C	0.42 ± 0.077	0.46 ± 0.074 *	0.54 ± 0.056 *&	0.69 ± 0.038 *&	0.83 ± 0.074 *&	0.92 ± 0.17 *&	1 ± 0.106 *&	1.08 ± 0.074 *&
	10 °C	0.39 ± 0.082	0.48 ± 0.095 *	0.59 ± 0.091	0.78 ± 0.038 *	0.94 ± 0.21	1.06 ± 0.075 *	1.12 ± 0.094 *	1.25 ± 0.186 *
	20 °C	0.4 ± 0.076	0.53 ± 0.049	0.63 ± 0.058	0.92 ± 0.091	1.12 ± 0.199	1.23 ± 0.058	1.28 ± 0.101	1.34 ± 0.097
MDA	4 °C	6.25 ± 0.27	5.61 ± 0.37 *&	8.37 ± 0.43 *	12.99 ± 0.79 *	10.92 ± 0.99 *&	11.84 ± 0.67 *&	14.52 ± 0.81 *&	16.12 ± 1.32 *&
	10 °C	6.25 ± 0.15	9.48 ± 0.77 *	8.57 ± 0.63 *	13.43 ± 0.74 *	13.67 ± 0.87 *	17.58 ± 1.00 *	18.03 ± 0.87 *	20.81 ± 0.79 *
	20 °C	6.25 ± 0.20	11.87 ± 0.59	12.41 ± 0.72	14.67 ± 0.81	18.45 ± 0.64	21.46 ± 0.84	22.43 ± 1.24	25.4 ± 1.42
PAL	4 °C	8.00 ± 0.47	7.5 ± 0.68	6.28 ± 0.44	5.75 ± 0.31	4.67 ± 0.31 *&	4.32 ± 0.49 *&	4.07 ± 0.21 *&	3.86 ± 0.48 *&
	10 °C	8.00 ± 0.25	7.24 ± 0.37	6.79 ± 0.44	6.18 ± 0.21	5.87 ± 0.48	5.53 ± 0.35	5.36 ± 0.35	4.83 ± 0.42
	20 °C	8.00 ± 0.31	7.08 ± 0.51	6.57 ± 0.48	6.35 ± 0.57	6.04 ± 0.33	5.4 ± 0.28	4.97 ± 0.59	4.65 ± 0.28
POD	4 °C	2.28 ± 0.29	3.21 ± 0.46 *	3.55 ± 0.27 *&	4.78 ± 0.63 *&	6.47 ± 0.76 *&	9.74 ± 1.06 *&	8.41 ± 0.74 *&	7.62 ± 0.69 *&
	10 °C	2.55 ± 0.34	3.54 ± 0.55	3.94 ± 0.59	6.36 ± 0.58 *	10.69 ± 0.68 *	12.54 ± 0.94 *	12.67 ± 0.53 *	10.48 ± 0.59 *
	20 °C	2.32 ± 0.29	3.87 ± 0.33	7.58 ± 0.67	12.72 ± 0.48	14.56 ± 1.25	15.76 ± 0.97	16.72 ± 1.02	17.46 ± 1.43
PPO	4 °C	13.05 ± 0.15	11.42 ± 0.22 *	10.32 ± 0.57 *&	8.75 ± 0.46 *	12.55 ± 0.87 *&	9.32 ± 0.94 *&	7.25 ± 0.64 *	6.45 ± 0.53 *
	10 °C	13.05 ± 0.08	11.42 ± 0.15 *	8.05 ± 0.74 *	9.00 ± 0.51 *	9.45 ± 0.58 *	14.75 ± 0.53 *	7.45 ± 0.72 *	6.85 ± 0.62 *
	20 °C	13.05 ± 0.19	14.32 ± 0.28	15.27 ± 0.82	16.15 ± 0.35	16.6 ± 1.04	17.4 ± 1.24	17.94 ± 0.55	18.42 ± 0.47

Means ± standard error (*n* = 3). *, compared to 20 °C, *p* < 0.05; &, compared to 10 °C, *p* < 0.05.

**Table 2:** Effects of different proportions of gas on changes in BD, MDA content, PAL activity, POD activity and PPO activity of Lanzhou lily bulbs.

Items	Gruops	0d	2d	4d	6d	8d	10d	12d	14d
BD	CK	0.88 ± 0.462	1.72 ± 0.372	2.10 ± 0.400	2.64 ± 0.502	2.98 ± 0.593	3.36 ± 0.383	3.72 ± 0.492	5.41 ± 0.723
	MAP1	0.88 ± 0.365	1.25 ± 0.271 *$+	1.56 ± 0.591 *$+	1.75 ± 0.503 *$+	1.94 ± 0.375 *&$+	2.08 ± 0.499 *&$+	2.17 ± 0.294 *+	2.23 ± 0.549 *$+
	MAP2	0.88 ± 0.275	1.33 ± 0.369 *$+	1.47 ± 0.306 *$+	1.59 ± 0.374 *$+	1.64 ± 0.586 *#	1.82 ± 0.428 *#	2.06 ± 0.618 *+	2.04 ± 0.370 *$+
	MAP3	0.88 ± 0.438	1.48 ± 0.452 *#&	1.77 ± 0.169 *#&	1.86 ± 0.265 *#&	2.01 ± 0.424 *#	2.17 ± 0.314 *#	2.25 ± 0.172 *+	2.31 ± 0.220 *#&
	MAP4	0.88 ± 0.390	1.52 ± 0.260 *#&	1.82 ± 0.311 *#&	1.97 ± 0.340 *#&	2.12 ± 0.310 *#	2.32 ± 0.590 *#	2.32 ± 0.429 *	2.43 ± 0.517 *#&
MDA	CK	1.68 ± 0.68	4.96 ± 0.37	9.73 ± 0.89	13.90 ± 0.23	17.18 ± 0.88	18.71 ± 0.58	21.52 ± 0.79	22.47 ± 0.98
	MAP1	1.68 ± 0.35	2.78 ± 0.45 *&$+	6.57 ± 0.47 *&$+	9.30 ± 0.31 *&$+	9.78 ± 0.51 *&$+	10.18 ± 0.48 *&$+	11.26 ± 0.87 *&+	12.36 ± 0.33 *&$+
	MAP2	1.68 ± 0.26	2.23 ± 0.25 *#+	3.96 ± 0.37 *#+	5.90 ± 0.53 *#&+	7.28 ± 0.25 *#&+	8.06 ± 0.62 *#&+	9.42 ± 0.77 *#&+	10.74 ± 0.54 *#+
	MAP3	1.68 ± 033	2.38 ± 0.45 *#+	3.85 ± 0.44 *#+	6.11 ± 0.21 *#+	8.40 ± 0.48 *&#+	9.13 ± 0.35 *&#+	10.52 ± 0.65 *&+	12.17 ± 0.42 *#+
	MAP4	1.68 ± 0.40	4.83 ± 0.47#&$	9.42 ± 0.68#&$	12.23 ± 0.44 *#&$	13.70 ± 0.31 *#&$	14.26 ± 0.31 *#&$	15.38 ± 0.49 *#&$	16.42 ± 0.82 *#&$
PAL	CK	15.929 ± 0.482	14.623 ± 0.429	13.228 ± 0.603	11.476 ± 0.445	10.819 ± 0.483	9.741 ± 0.329	9.007 ± 0.798	7.792 ± 0.535
	MAP1	15.929 ± 0.577	14.149 ± 0.583	12.707 ± 0.389	9.574 ± 0.886 *$+	9.366 ± 0.742 *	8.64 ± 0.528 *&$+	8.136 ± 0.426&$+	7.128 ± 0.667&$
	MAP2	15.929 ± 0.672	13.995 ± 0.378	12.667 ± 0.488	9.954 ± 0.879 *+	8.778 ± 0.973 *	7.42 ± 0.902 *#	6.369 ± 0.519 *#&	5.955 ± 0.385 *#+
	MAP3	15.929 ± 0.218	14.372 ± 0.462	12.642 ± 0.672	10.402 ± 0.489#&	8.739 ± 0.767 *	7.768 ± 0.484 *#	7.247 ± 0.619 *#&	6.329 ± 0.449 *#+
	MAP4	15.929 ± 0.569	14.282 ± 1.003	13.146 ± 0.953	10.899 ± 0.731#&	9.366 ± 0.450 *	8.105 ± 1.252 *#	7.477 ± 0.512 *#$	7.277 ± 0.930&$
POD	CK	5.929 ± 0.362	8.623 ± 0.672	9.228 ± 0.489	14.476 ± 0.367	16.819 ± 0.567	15.741 ± 0.672	14.007 ± 0.378	13.792 ± 0.488
	MAP1	5.929 ± 0.569	8.149 ± 1.003	8.707 ± 0.886	9.574 ± 0.953 *&+	9.366 ± 0.731 *$&+	6.64 ± 0.450 *&+	9.636 ± 0.252 *&+	9.128 ± 0.512 *&$
	MAP2	5.929 ± 0.484	7.995 ± 0.619 *	6.667 ± 0.449 *#$+	5.954 ± 0.472 *#$+	5.778 ± 0.282 *#$+	5.42 ± 0.429 *#$+	7.369 ± 1.003 *#$	6.955 ± 0.445 *#+
	MAP3	5.929 ± 0.426	8.372 ± 0.273	8.642 ± 0.902	9.402 ± 0.519 *&+	7.739 ± 0.385 *#&	7.105 ± 0.218 *&+	9.847 ± 0.390 *&+	7.329 ± 0.250 *#+
	MAP4	5.929 ± 0.577	8.582 ± 0.583	9.146 ± 0.289	12.899 ± 0.879 *#$&	8.366 ± 0.483 *#&	7.768 ± 0.329 *#$&	7.877 ± 0.298 *#$	9.577 ± 0.335 *&$
PPO	CK	10.929 ± 0.473	13.623 ± 0.402	10.228 ± 0.219	9.741 ± 0.385	11.476 ± 0.218	10.819 ± 0.390	8.007 ± 0.250	7.128 ± 0.426
	MAP1	10.929 ± 0.369	13.149 ± 0.303 *&	9.707 ± 0.686 *&	8.792 ± 0.253 *&	10.574 ± 0.531 *&	9.369 ± 0.450 *#$	8.768 ± 0.252 *&$+	6.64 ± 0.512 *&
	MAP2	10.929 ± 0.484	11.795 ± 0.219 *#+	9.146 ± 0.449 *#	7.636 ± 0.772 *#$+	9.778 ± 0.282 *#$+	8.366 ± 0.429 *#+	6.955 ± 0.203 *#	5.877 ± 0.445 *#+
	MAP3	10.929 ± 0.262	12.582 ± 0.272 *	9.642 ± 0.489 *	8.954 ± 0.173 *&	10.105 ± 0.567 *&	7.847 ± 0.772 *#+	7.329 ± 0.378 *#	6.402 ± 0.488 *
	MAP4	10.929 ± 0.377	12.672 ± 0.583 *&	9.667 ± 0.689 *	8.739 ± 0.379 *&	10.899 ± 0.483 *&	9.366 ± 0.129 *#$	7.577 ± 0.298 *#	6.842 ± 0.335 *&

Means ± standard error (*n* = 3). *, compared to CK, *p* < 0.05; #, compared to MAP1, *p* < 0.05; &, compared to MAP2, *p* < 0.05; $, compared to MAP3, *p* < 0.05; +, compared to MAP4, *p* < 0.05.

## Data Availability

The data used to support the findings of this study can be made available by the corresponding author upon request.

## Supplementary Materials

The following supporting information can be downloaded at: https://www.mdpi.com/article/10.3390/foods12061335/s1, Supplementary Material S1: Data quality assessment.xlsx; Supplementary Material S2: DEGs.xlsx; Supplementary Material S3: DEGs-GO analysis.xlsx; Supplementary Material S4: DEGs-kegg enrichment.xlsx; Supplementary Material S5: DMs.xlsx; Supplementary Material S6: DMs-kegg enrichment.xlsx.

## Abbreviations

4CL	4-coumarate:CoA ligase
ACAA1	Acetyl-CoA acyltransferase 1
ADH	Alcohol dehydrogenase
ANS	Anthocyanidin synthase
AOC	Allene oxide cyclase
BD	Browning index
BP	Biological process
C4H	Cinnamic acid 4-hydroxylase
CAD	Cinnamoyl alcohol dehydrogenase
CC	Cellular component
CCR	Cinnamoyl CoA reductase
CHI	CHS-chalcone isomerase
CHR	Chalcone reductase
CHS	Chalcone synthase
COMT	Caffeic acid O-methyltransferase
CWC	Chinese water chestnut
DEGs	Differentially expressed genes
DFR	Dihydroflavonol 4-reductase
DMs	Differential metabolites
EUG	Eugenol emulsions
F3′H	Flavanone 3′-hydroxylase
F3H	Flavanone 3 hydroxylase
F5H	Flavanone 5 hydroxylase
FFV	Fresh-cut fruits and vegetables
HCT	Hydroxycinnamoyl transferase
HPCD	High-pressure carbon dioxide
HPL1	Hydroperoxide lyase l
LOX	Lipoxygenase
MAP	Modified atmosphere packaging
MDA	Malonaldehyde
MF	Molecular function
MFP2	Enoyl-CoA hydratase/3-hydroxyacyl-CoA dehydrogenase
OPLS-DA	Orthogonal partial least-squares discriminant analysis
OPR	12-oxo-phytodienoic acid reductase
PAL	Phenylalanine ammonia lyase
PCA	Principal component analysis
PLA2G	Phospholipase A2 Group
POD	Peroxidase
PPO	Polyphenol oxidase
PVP	Polyvinylpyrrolidone
ROS	Reactive oxygen species
